# Supplementary material for: What does it take to learn the rules of RNA base pairing? A lot less than you may think
Source: bioRxiv. 2025 Aug 2:2025.07.31.668042. Preprint. [Version 1] doi: 10.1101/2025.07.31.668042 (PMC12324431; doi:10.1101/2025.07.31.668042)
Supplement: Supplement 1 [file media-1.gz › supplemental_material/src/rscape_v2.6.0.tar.gz!/rscape_v2.6.0/lib/infernal/Manuscripts/inf-1-appnote/figs/roc.pdf]

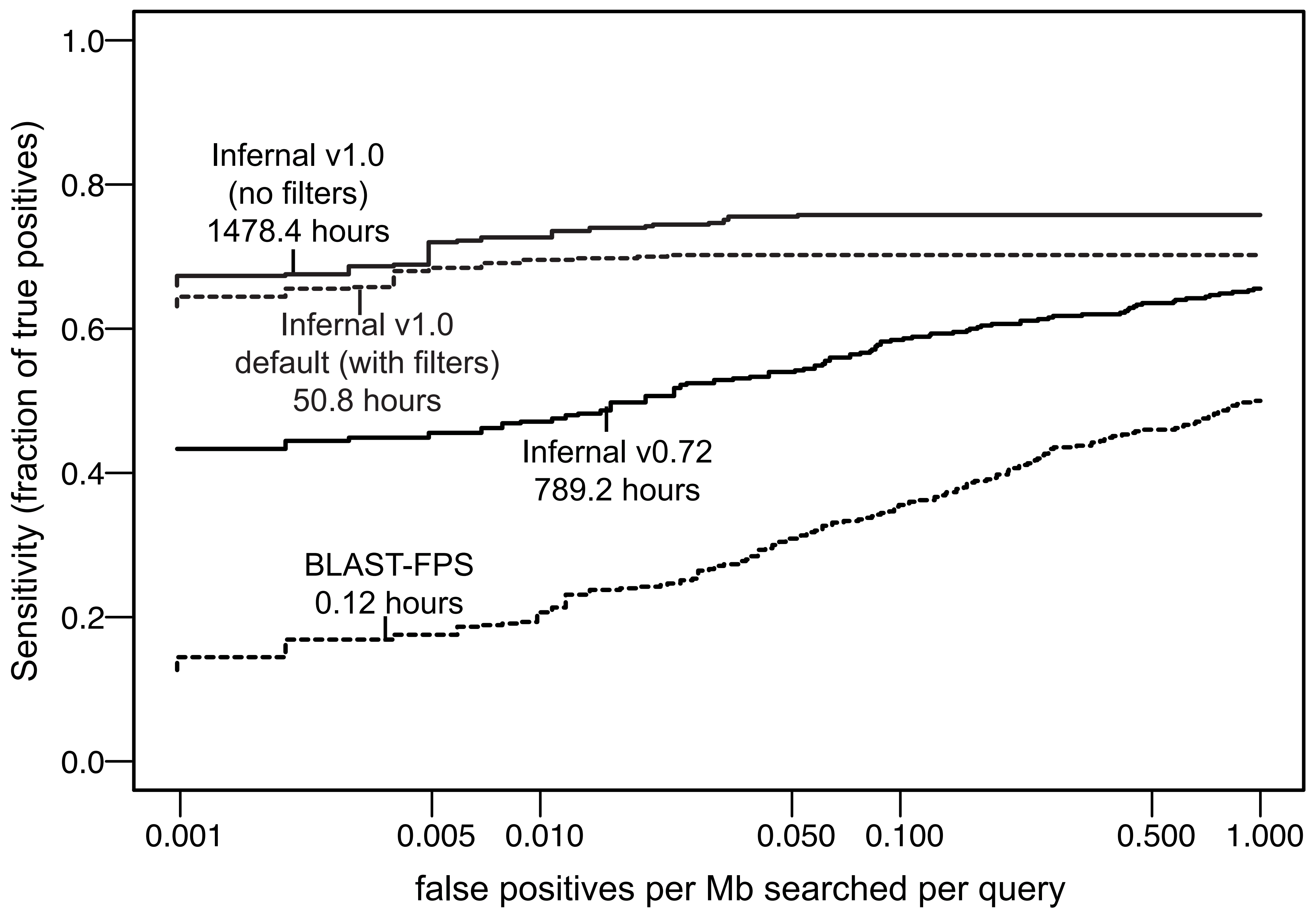

Sensitivity (fraction of true positives)
1.0
0.8
0.6
0.4
0.2
0.0
Infernal v1.0
(no filters)
1478.4 hours
Infernal v1.0
default (with filters)
50.8 hours
Infernal v0.72
789.2 hours
BLAST-FPS
0.12 hours
0.001
0.005
0.010
0.050
0.100
0.500
1.000
false positives per Mb searched per query
